# A review of the evidence for a protective role of uric acid in Parkinson’s disease

**DOI:** 10.1038/s41531-025-01169-8

**Published:** 2025-11-19

**Authors:** Hanxiang Liu, Gavin P. Reynolds

**Affiliations:** 1 Faculty of Life Science and Technology, Kunming University of Science and Technology, Kunming, China; 2 Medical School, Kunming University of Science and Technology, Kunming, China; 3 Southwest United Graduate School, Kunming, China; 4 Department of Rehabilitation Medicine, The Third People’s Hospital of Yunnan Province, Kunming, China; 5 Biomolecular Sciences Research Centre, Sheffield Hallam University, Sheffield, UK

**Keywords:** Diseases of the nervous system, Parkinson's disease, Neuroscience, Pathogenesis, Risk factors

## Abstract

The relationship between the circulating antioxidant uric acid (UA) and Parkinson’s disease (PD) has attracted much interest. This review of the evidence indicating whether UA may have a protective role in the development and/or progression of PD draws on the findings of reduced serum UA in PD, critically assesses the more equivocal genetic results, and discusses experimental observations that UA may provide protection against the pathogenic mechanisms of PD.

## Uric acid in the human body

Uric acid (UA) is, in humans, the end product of purine metabolism and plays an important role in the human body. While in many mammalian species UA is further degraded to allantoin, in humans and certain other primates the uricase activity responsible for this metabolic removal has been lost. The evolutionary advantage this provides is not fully understood but may in part relate to its neuroprotective properties discussed below, although it comes at a substantial cost associated with the incidence of, for example, gout and cardiovascular disease^1^. Certainly, there is now strong evidence not only for a causal effect on gout but also for associations with metabolic syndrome and related cardiometabolic disorders^2^.

The majority of circulating UA comes from the metabolism of endogenous purine nucleotides in the liver, while approximately 20% originates from the diet^3^. A series of transporters primarily acting in the kidney is responsible for the control of UA in the blood^3^, with URAT1 (coded by SLC22A12) responsible for most kidney reabsorption into the blood and ABCG2 (or breast cancer resistance protein, BCRP) mainly involved in secretion into urine. Additional organic anion transporters also contribute, while a further important transporter is GLUT9 (coded by SLC2A9); variation in this gene makes the greatest contribution to serum UA in both European and non-European populations^4^. Thus while UA can be influenced by environmental, notably dietary, factors, serum UA is considered to be mainly genetically determined. A large study of the genetic factors contributing to serum UA has identified 183 loci, but still only explains about 7.7% of the variance^5^.

These complex control mechanisms contributing to UA homoeostasis reflect its importance in the human body. An under-researched area is the role of these transporters in the brain—essential to understanding the influence of UA on neurodegenerative processes—which will be discussed later in this article. UA is considered the major circulating antioxidant and thus provides protection from oxidative stress and consequent damage; it also has ion-chelating properties^6^. In the context of brain function, these may provide protection from neurodegenerative processes. However, the functional in vivo activity of UA’s antioxidant properties remain unclear, with possible pro-oxidative effects also being proposed^3^. Furthermore any consideration of the protective action of UA in the central nervous system needs to be seen in the light of its well-established contributary role in the development of peripheral cardiovascular and metabolic diseases^2,3^.

## Uric acid in Parkinson’s disease pathogenesis

There has been much interest in the potential role of UA in Parkinson’s disease (PD). PD is characterised by a progressive loss of, primarily, dopaminergic neurons of the substantia nigra that project to the striatum, resulting in its motor symptoms of bradykinesia, rigidity and tremor. Other neuronal systems are also affected, resulting in non-motor symptoms including cognitive decline. The aetiology of PD is complex; most cases appear to be sporadic, in which both genetic and environmental risk factors play a role^7^, while a small proportion arise from monogenic mutations. PD and several related neurodegenerative disorders can also been considered as proteinopathies, in which the core neurodegeneration of nigrostriatal dopaminergic neurons in PD is also associated with a more widespread cellular deposition of alpha-synuclein^8^. These deposits disrupt the structure and function of cells^9^; the aggregating alpha-synuclein also binds preferentially to mitochondria where it can disrupt energy metabolism leading to oxidative stress.

In both of these inter-related pathologies of PD – dopaminergic neurodegeneration and synucleinopathy – UA has been implicated. This has mainly derived from experimental studies, although the first results emerged over 30 years ago with the observation of reduced UA in the substantia nigra of PD patients at post mortem^10^. This finding brought together the antioxidant property of UA with the consistently topical concept of oxidative stress as a critical factor in the pathogenesis of PD. It is well established that the chemical environment of the substantia nigra in PD is indicative of oxidative damage^11^. Thus a reduction in a protective antioxidant such as UA may result in increased oxidative stress and subsequent damage. A further theory relates to the ability of UA to form complexes with metal ions such as Fe(III)^6^; a relative increase in ferric ions may contribute to the pathological process in the substantia nigra^11^. However, whether the reduction of UA in the PD substantia nigra is causal, a consequence or an epiphenomenon related to other chemical processes in the PD brain remains unresolved.

## Clinical studies of uric acid in Parkinson’s disease

What has primarily driven research into the relationship between UA and PD are epidemiological studies of serum UA and its related genetic biomarkers. Investigations of blood UA have consistently indicated associations between reduced UA and PD, confirmed in a recent meta-analysis of cohort studies^12^. This analysis provides a useful overview of 18 studies, and also demonstrates that male sex and younger age may be associated with a stronger protective effect of UA. This is interpreted as possibly reflecting increased exposure to neurotoxic chemicals in men and in the work force. Further support comes from cross-sectional studies comparing PD and matched control subjects^13^, while others have investigated the role of UA in disease progression and severity, with findings suggesting an influence of low UA in each of these clinical factors^14,15^.

Gout, an inflammatory arthritic disorder which is the consequence of deposits of UA crystals, is the archetypal hyperuricemic disease and provides a model to investigate the relationship between blood UA and brain disease. Results are, however, inconsistent; while studies have shown that there may be a protective influence of gout on PD^16^, others show this to be restricted to an older cohort^17^. Furthermore, a recent meta-analysis of 10 studies concluded that there was no significant effect of gout on the risk of developing PD^18^. Another meta-analysis indicated that while there was strong evidence for elevated serum UA as a protective factor for PD, the influence of gout on PD is far weaker^19^. Reconciling these findings is not straightforward but may relate to two arguments. One is that the loss of a neuroprotective effect of UA when it is reduced below normal is not necessarily modelled by comparing serum levels in unaffected subjects with the hyperuricemia associated with gout. Such results may also be confounded by effects of UA-lowering medication and dietary changes. The other argument is that gout is primarily an inflammatory disease resulting in systemic increases in proinflammatory factors^20^; these may cross the blood-brain barrier to promote neurodegenerative processes.

CSF has substantially lower concentrations of UA than are found in blood, although the two are correlated^21^. A large longitudinal study has shown that CSF UA is relatively higher in PD patients with a slower clinical progression^22^, consistent with findings from blood samples.

There is evidence that the reduction of UA in PD might reflect an increase in its metabolic removal, with elevated serum concentrations of allantoin, its immediate oxidative metabolite^23^. This finding, more pronounced in patients with autonomic dysregulation, is interpreted as indicative of increased oxidative stress and therefore may represent an epiphenomenon providing evidence for reverse causality in the UA-PD relationship. Furthermore there is also some limited and inconsistent evidence that changes in transporter activity may contribute to the reductions in UA seen in PD and these are reviewed below.

## Genetic evidence for the role of uric acid in Parkinson’s disease

While, as mentioned, UA derives from the metabolism of purine and hence will be influenced by diet, serum UA is primarily related to genetic factors, particularly those associated with UA transport. This has been a concern in relation to high concentrations of UA, which are well established as contributing to risk of gout as well as cardiovascular disease. However, findings from these genetic risk factors for disease associated with high UA have been applied to identifying risk genes for low UA in PD. While this has some inherent logic, it has not been fully explored whether it is valid to assume a linearity of the relationship between gene and serum UA in which genetic polymorphisms differentiating normal and high UA associated with, say, risk of gout are the same as those differentiating normal and low UA associated with risk of PD. Certainly, there seems to be a mismatch between the influence of high UA and that of gout in risk of PD, as mentioned above.

However, there is now a substantial literature of genetic association studies of UA-related genes and PD. Most studies have focused on the UA transporter genes ABCG2 and SLC2A9; a meta-analysis has demonstrated that single nucleotide polymorphisms (SNPs) in these genes can affect plasma UA concentrations^24^. These authors make the point that genetic effects often appear to be greater in males, perhaps related to their tendency to higher UA concentrations. This is notable in the light of the observation from cohort studies that the association of serum UA with PD is also stronger in males^12^. The genetic variants then provide the opportunity to investigate whether there is an association between genetically-determined serum UA and PD. An early study^25^ addressed this question by genotyping 9 SNPs associated with serum UA in a series of PD and matched control subjects to obtain a polygenic risk score (PRS) for low UA. It was found that those with a high PRS had a greater risk of having PD than those with low PRS. Other similar studies have been less supportive of the genetic UA-PD relationship. Hughes et al.^26^ found, among genes related to UA transport, only SLC2A9 to have SNPs associated, weakly, to UA levels; they did not find this genetic factor to associate with PD risk.

Genetic factors associated with serum UA can be used as an explicit proxy for serum UA concentration, permitting the assessment of its potential causal relationship with PD, a technique referred to as Mendelian randomisation (MR). Another early study^27^ employed three SLC2A9 SNPs to identify a genetic proxy for serum UA concentration; this was found to have a significant effect on progression to requiring treatment in early PD. This causal evidence was, however, not supported by a population study using SLC2A9 and ABCG2 SNPs; despite a significant association of plasma UA with PD incidence, the genetic association with PD was not apparent^28^. Several further studies also fail to identify such potentially “causal” relationships between UA-related genes and PD; a recent example confirms the relationship of PD with serum UA but shows no genetic association in the same sample^29^. Furthermore, while the positive study of Simon et al.^27^ specifically addressed progression within PD and other MR investigations primarily studied disease association, the most comprehensive MR study did not identify a protective effect on either risk or progression of PD^30^.

The conclusions from these essentially negative MR findings have, however, been questioned in a well-argued critique^31^, pointing out the limitations of genetic markers that explain only a small component (i.e., 7.7% at most^5^) of serum UA variance and querying whether the core assumptions of MR, including the absence of confounds as well as a strong association with UA, are met. We would further suggest that the approach is invalidated by the violation of a particular requirement of MR studies, i.e. that that the genetic instrument can only influence the outcome through its effect, in this case, on serum UA. The genetic correlates relate primarily to variants in the genes coding for transporters controlling plasma UA, yet such presumed functional variants will also influence, through the same proteins, the transport of UA into the brain and subsequently into neurons and glia, as described below. It is these poorly-understood processes involved in the transport of UA that are particularly important in determining the disposition and action of UA within the brain, but are unaccounted for in the assumptions of the MR studies.

Furthermore, the relatively small genetic influence on serum UA implies a greater contribution from other factors, perhaps those environmentally mediated. In this respect, it is relevant that there is functional variation in DNA methylation of UA-controlling genes, most notably including SLC2A9^32^. These authors reported that such epigenetic factors could explain 11.6% of the variance in serum UA, substantially greater than the 7.7% explained by genetic variability alone. This finding indicates that epigenetic variation, which may well be susceptible to contextual and other environmental influences, has a greater effect on serum UA than the identified genetic factors and certainly deserves further investigation in PD.

## Experimental studies—in vivo

Given that SLC2A9 contributes to UA blood levels through its activity in the kidney, other processes inevitably mediate the influence of serum UA on the risk or progression of a brain disease such as PD. This will include its uptake into the brain, likely to be limited by the blood-brain barrier (BBB). Once in the brain, there are several factors that might be involved in the action of UA to inhibit the neurodegenerative process. These include its transport into specific cells and its particular neuroprotective action—of which an antioxidant effect in dysfunctional neurons and an anti-inflammatory effect have strong supportive evidence from experimental studies, as discussed below. Inflammation is a consequence of alpha-synuclein aggregation via an activation of microglia^9^, which, in a prolonged inflammatory state, can result in neurodegeneration. Oxidative free radical production is a process central to the neurotoxicity of inflammation; neurodegeneration can stimulate further inflammatory activity, resulting in further neuronal damage and death. Thus, the three mechanisms of oxidative stress, inflammation, and alpha-synuclein aggregation are intimately interrelated; such inter-relationships will also apply to any effects of UA on these neurotoxic mechanisms (Table 1).

**Table 1:** Some proposed mechanisms of neuroprotection by uric acid

*Mechanism*	*Model system*	*reference*
Reducing oxidative stress:		
Increased antioxidant enzymes	in vivo (mouse)	35
Increased glutathione	in vivo (mouse) in vitro (astrocytes) in vitro (neurons)	35 52 39
Increased Nrf2 signalling	in vivo (mouse) in vitro (neurons) in vitro (astrocytes)	35 39 52
Reducing inflammation:		
Decreased pro-inflammatory cytokines	in vivo (mouse) in vitro (microglia) in vivo (rat)	35 36
Decreased astrocyte activation	in vitro (astrocytes)	52
Decreased microglial activation	in vitro (microglia) in vivo (rat)	36
Reducing synucleinopathy:		
Decreased inter-neuronal transmission of alpha-synuclein	in vivo (mouse) in vitro (neurons)	40
Decreased accumulation of alpha-synuclein	in vivo (mouse) in vitro (neurons)	41

Animal models attempting to replicate the dopaminergic pathology and clinical features of PD have been critical to our understanding of the pathogenic mechanisms of the disease. However, investigation of the effect of UA on such models is limited by the profound differences between humans and e.g., rodents in UA metabolism, primarily due to the loss in humans of a functional uricase. This hepatic enzyme converts UA to allantoin, and its absence results in serum UA concentrations relatively elevated by, typically, an order of magnitude. The consequences of this difference have been recognised in the investigation of the toxic mechanisms associated with gout, where the concentrations of UA that induce peripheral microcrystallisation and its related inflammatory reaction are not easily reached in rodents. This limitation has been addressed by increasing serum UA in various ways, both pharmacological and genetic, including knockout of the uricase gene.

An important early example investigated the effect of both knockout and transgenic uricase in mice receiving intracerebral 6-hydroxydopamine administration, which leads to dopaminergic neurodegeneration^33^. The increase in UA following uricase knockout resulted in attenuation of behavioural and neurochemical pathology, while animals overexpressing this gene showed an enhancement of these measures. This valuable and informative approach appears not to have been explored further in the context of PD models, although the recent report of a rat knockout model^34^ should extend the opportunity to do so.

Nevertheless, there are studies providing evidence for an influence of circulating UA on the pathology and behaviour of animal models of PD independent of any “humanising” effects on uricase activity. Another Parkinsonian model employing 1-methyl-4-phenyl-1,2,3,6-tetrahydropyridine (MPTP) toxicity found that UA could reduce oxidative stress, inflammation, and dopaminergic neuronal dysfunction in the brain^35^. UA also induced an increase in Nrf2, a transcription factor that regulates genes involved in antioxidant response^35^. A further, if less specific, model of dopaminergic degeneration can be produced in the rat with the inflammatory toxin lipopolysaccharide (LPS); this too could be attenuated by UA^36^.

## Experimental studies—in vitro

Cellular model systems, while further removed from the clinical context, have shed light on the molecular mechanisms of UA’s likely neuroprotective action. An early study demonstrated a protective effect of UA on spontaneous death of dopaminergic neurons in culture, with an antioxidant mechanism targeting Fe-produced reactive oxygen species^37^. The protective effect of UA in parkinsonian model systems is also found to be dependent on Glut9-mediated transport, which is upregulated by UA in dopaminergic neurons in vitro^38^. However, whether the neuroprotection occurs primarily within the dopamine neuron^38^, at extracellular^37^ sites, or in glia remains unresolved. Bao et al.^36^ showed that the neurotoxic activation of microglia with LPS can be reduced in the presence of elevated UA, an effect shown to be mediated by cellular UA uptake. This neuroprotective mechanism was found to occur in concert with a suppression of proinflammatory cytokines released from the activated microglia, suggesting an anti-inflammatory mechanism upstream from the neurodegeneration of dopaminergic neurons. As also observed in vivo^35^, the neuroprotective action of UA on dopaminergic neurons in culture has effects on signalling by Nrf2^39^.

There is further experimental evidence implicating UA as a protective factor in the intraneuronal deposition of alpha-synuclein. A recent study^40^ showed UA to regulate the transmission of alpha-synuclein in both animal and cellular Parkinsonian models, with a reduction in dopaminergic cell damage. This finding is consistent with other reports^41^ demonstrating UA-induced increases in autophagy and reduction in alpha-synuclein accumulation.

## Transport and disposition of uric acid in the brain

The concentrations of UA are, as mentioned, substantially lower in the CSF than in peripheral blood, indicating that UA does not rapidly equilibrate across the BBB and that there may be active transport mechanisms influencing CNS UA. There is strong evidence for the latter; human post mortem studies show both GLUT9 and URAT1 are found in the choroid plexus and the former is also found in brain ependymal cells that contribute to the BBB^42^. The position of GLUT9 on the apical side of the epithelial cells of the choroid plexus suggests its involvement in direct transport of vascular UA into the CNS. URAT1 is found on the basolateral membrane of these epithelial cells^42^, which suggests these two proteins are functioning in concert as transcellular UA transporters, as they do to achieve resorption in the kidney. ABCG2 is also expressed along with GLUT9 in brain capillaries and epithelial cells of the choroid plexus in mouse brain^43^ and in human brain microvessels^44^. Thus, all three of the main UA transporters may contribute to the brain concentrations of UA through their action at the blood-brain barrier, although ABCG2 is thought to have the greatest effect, probably acting to remove UA from brain to blood^45^.

Gene expression studies in the mouse brain have demonstrated the presence of GLUT9 in many regions, notably including the hippocampus and cortex^46^. These authors showed distinct cellular expression in most layers of the neocortex along with specific and selective staining in the dentate gyrus and CA regions of the hippocampus, as well as in the Purkinje cell layer of the cerebellum. While not confirmed by specific neuronal markers, the selectivity of staining is indicative of neuronal expression, a conclusion confirmed by immunostaining of mouse periventricular neurons^43^. Neuronal dopaminergic cell lines have also demonstrated the presence of GLUT9 and its up-regulation in response to UA^38^. ABCG2, while important in blood-brain barrier transport of UA and other molecules, is not reportedly found on human neurons^47^. However, it is present in cortical glial cells, where it may be upregulated in amyotrophic lateral sclerosis^48^. There are also indications of an upregulation of ABCG2 in correlation with an inflammatory marker in human brain tissue^49^. Furthermore, any situation in which energy metabolism in the brain is compromised may well influence the expression and function of UA transporters^45^. These observations, reflecting regulatory effects on the transcriptional control of transporters affecting brain UA, demonstrate tissue-specific effects on UA activity, which further question the validity of MR studies discussed above.

The cellular distribution of UA and its transporters within the brain has otherwise been little studied, although we can get some clues from the investigation of peripheral cells. Human monocytes can take up UA via GLUT9 transport, on which the anti-inflammatory effect of UA is dependent^50^. Soluble UA can reduce the number of monocytes positive for pro-inflammatory cytokines IL-6 and TNF-alpha^35^. While the pro-inflammatory effect of microcrystalline UA is well-established^3,20^, there are several indications that soluble UA free of UA crystals has protective effects against inflammatory mechanisms and toxicity^50^ with effects on macrophage polarisation towards the anti-inflammatory M2 phenotype^51^. It seems likely that these observations in macrophages may well extend into the microglia^36^, their equivalent cells in the CNS. Others have identified astrocytes as a target for the neuroprotective effects of UA, demonstrating effects on astrocytic glutathione and Nrf2, and providing further support for the involvement of UA in antioxidative processes^52^. UA effects on astrocyte function are also indicated by its ability to protect against the peroxide-induced toxicity of rodent dopaminergic cells in vitro via an effect on astrocytes^53^.

## Uric acid as a pharmacotherapy in Parkinson’s disease

A clinical intervention study, in which a systemic increase in UA is brought about by a pharmacological agent, might be considered the ultimate test of UA as a potential protective factor. Attempts to increase exposure to UA have been made previously in the context of ischaemic stroke, one study finding that fewer patients underwent early ischaemic worsening after intravenous UA administration^54^. Such intervention may be valuable in the treatment of acute conditions, but infusion of large quantities (1000 mg) of UA is not a feasible therapeutic approach for chronic neurodegenerative disease. One alternative pharmacotherapy has been to administer inosine, a purine that is converted in the body to UA.

Following assessment of its feasibility, a randomised controlled trial of inosine was undertaken in PD patients^55^. The patient group was selected as having both relatively reduced serum UA and imaging evidence of dopaminergic deficits without yet requiring dopaminergic therapy. Despite inosine inducing a substantial increase in serum UA, no significant reduction in the rate of disease progression was observed.

These results appear clear-cut; the pharmacological elevation of serum UA does not result in any reduction in the progression of PD. Nevertheless, the authors do point out that they cannot rule out a positive effect on a small subgroup of patients, or an effect of UA occurring earlier in the neurodegenerative process. This concern is a common theme in neurodegenerative disease treatment, when disease-modifying therapies are often considered to be effective only, or mainly, in the early stages of the degenerative process. In this trial, the patients studied at 1–3 years post diagnosis were likely to have lost the majority of nigral and striatal dopaminergic function^56^. So, any protective effect of UA on the emergence of PD does not apparently extend to reducing disease progression in diagnosed patients.

A factor—be it genetic, environmental, or life-style—which contributes to a cellular environment protective against the development of PD is not necessarily going to protect against progression of the established disease. An analogy can be found in the influence of tobacco smoking. This has strong evidence demonstrating a causal protective effect on risk of PD^57^; while an intervention study of smoking would not be feasible, a retrospective study of cigarette smoking in PD indicates no effect on the progression of established disease^58^.

## Conclusions

In this article, we have attempted to review the evidence relating to the putative role of UA in PD. In drawing on studies that extend from clinical epidemiology, through genetics, pharmacotherapy and animal models, to cell biology and biochemistry, we have aimed to provide a comprehensible and accessible narrative, although this comes at the expense of a fully comprehensive and systematic (and inevitably longer) analysis. There remains much that is disputed, and some obvious lacunae in our understanding of the action of UA in the human brain. But we can draw some clear conclusions.

**Fig. 1:**
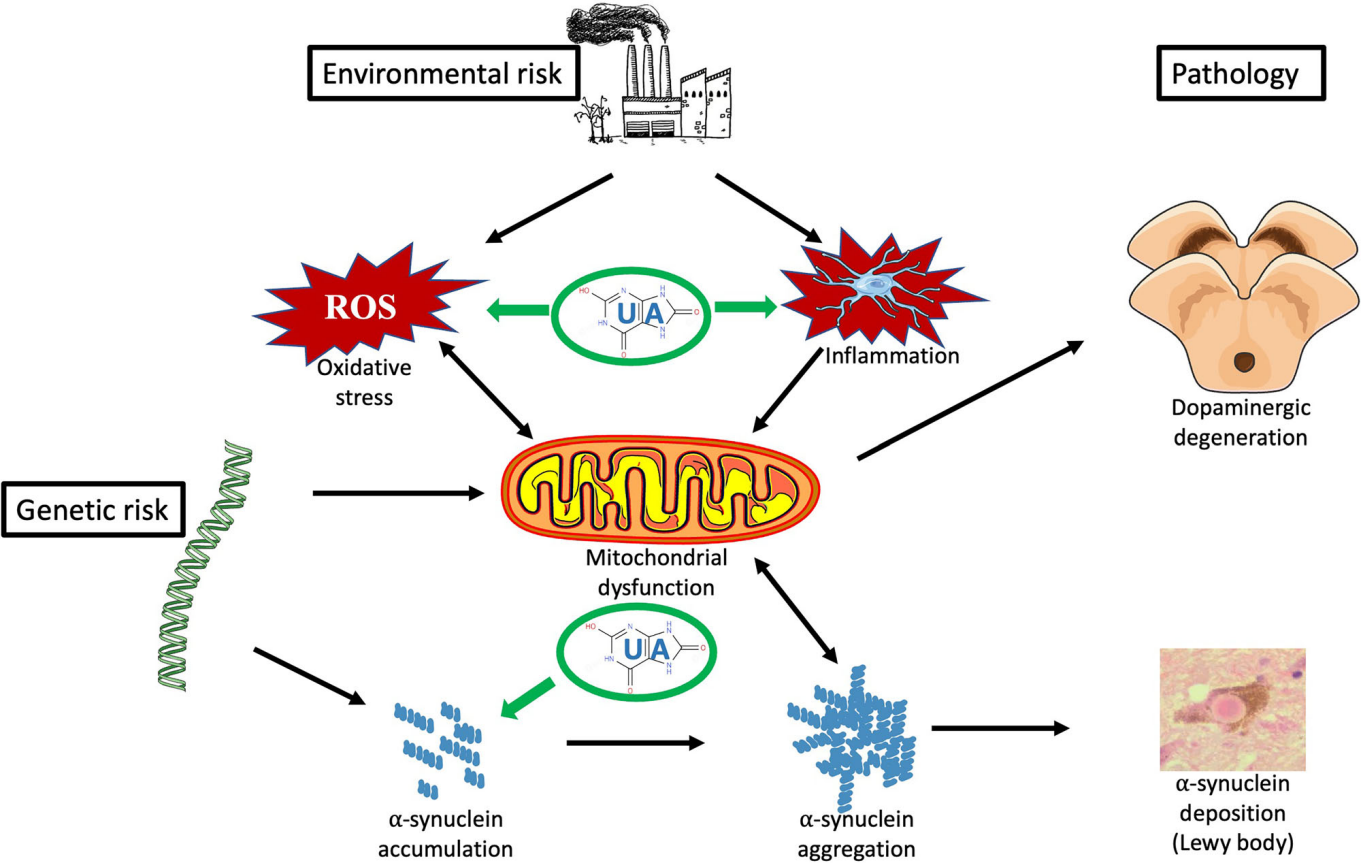
The pathogenic process in Parkinson’s disease and proposed protective sites of action for uric acid.

There is a very strong body of experimental evidence indicating a neuroprotective role of UA, mediated through anti-inflammatory and antioxidative mechanisms that themselves are likely to be closely interrelated (summarised in Fig. 1). This provides supportive, if circumstantial, evidence for a role for UA in protecting against the neuronal degeneration underlying PD. This conclusion is also based on a large number of clinical studies relating lower levels of serum UA to increases in PD prevalence or progression. Some contrary conclusions derive from using genetic markers as a proxy for circulating concentrations of UA; several of these MR studies indicate no significant relationship of PD with genetic correlates of serum UA. These findings are open to criticism in that, by ignoring the possible genetic effects on UA transport into and within the brain, the approach used violates a central assumption of such studies. However, an intervention study clearly demonstrates the lack of influence of an increase in serum UA on the progression of established PD. Identifying subjects earlier in the disease course might provide the opportunity for testing the efficacy of an earlier UA intervention when the majority of dopaminergic neurons remain intact.

Any effect of serum UA on the development or progression of PD needs to be considered in the light of other PD risk factors. This has been done for some of the stronger genetic PD risk factors (e.g. Koros et al.^59^) without clear evidence of an interaction effect, although a study exploring a genome-wide association database did identify an interaction of one risk SNP with UA^60^. For environmental risk, there appear to be no reports searching for interaction with UA. We found that reduced UA and agricultural work, proxy for increased exposure to pesticides, were both associated with PD in the same cohort^13^, although the study was not designed to identify interaction effects. This would seem to be a potentially fruitful area for further research since the major environmental risk factors for PD—exposure to pesticides, trichloroethylene, and air pollution—are likely to have their effects on dopaminergic neurodegeneration via toxic effects of oxidative stress and inflammation^61^.

Thus, we can clearly identify areas of research that deserve further study. At present, we still have a very limited knowledge of the transport processes for UA into and within the human brain. Determining the roles of, particularly, ABCG2 and GLUT9 in glia and neurons, how they respond to inflammatory challenge, to cellular activation, and to variation in UA concentrations is essential to understanding the disposition and activity of UA in the CNS. A reliance on animal experiments may be misleading, given the substantial changes, not only in UA metabolism but also in its transport, associated with primate evolution^62^. Nevertheless, greater use of uricase knock-out animals and similar “humanising” paradigms may prove valuable in studying the interaction of UA with clinically relevant models of PD neurodegeneration.

The observation that DNA methylation can account for rather more of the variance in serum UA than genetic polymorphisms^32^ indicates the strong potential of investigation into this and other epigenetic factors in relation to risk of PD and, of course, other disorders associated with UA. Sex-related effects too deserve attention here, particularly in the light of a very recent clinical study of DNA methylation associated with UA concentration. Intriguingly, it was found that males, but not females, showed an UA-related enrichment of methylation sites in genes involved with neuroprotective pathways^63^. Finally, it is important to acknowledge the similar protective role that UA is proposed to have in Alzheimer’s disease, indicating an influence not restricted to the dopaminergic neurodegeneration and synucleinopathy in PD. Thus, an improved understanding of the regulation of UA and its neuroprotective action in the human brain will shed further light on neurodegenerative processes and potentially identify novel targets for pharmacotherapeutic intervention.

## Data Availability

No datasets were generated or analysed during the current study.
